# Psychosocial impact of sickle cell disorder: perspectives from a Nigerian setting

**DOI:** 10.1186/1744-8603-6-2

**Published:** 2010-02-20

**Authors:** Kofi A Anie, Feyijimi E Egunjobi, Olu O Akinyanju

**Affiliations:** 1 Brent Sickle Cell and Thalassaemia Centre, Imperial College School of Medicine, Central Middlesex Hospital, London, NW10 7NS, UK; 2 National Sickle Cell Centre, Idi Araba, Lagos, Nigeria

## Abstract

Sickle Cell Disorder is a global health problem with psychosocial implications. Nigeria has the largest population of people with sickle cell disorder, with about 150,000 births annually. This study explored the psychosocial impact of sickle cell disorder in 408 adolescents and adults attending three hospitals in Lagos, Nigeria. A questionnaire was designed for the study, with some of commonly described areas of psychosocial impact including general public perceptions and attitudes, education, employment, and healthcare issues, and emotional responses.

The majority of participants thought that society in general had a negative image of SCD, and reported negative perceptions and attitudes. Some issues in education, employment, and healthcare were expressed, however these were in the minority of cases. The results also showed that depressive feelings were experienced in almost half the study population, even though feelings of anxiety or self-hate were uncommon. Clinical implications of these findings are considered.

## Introduction and Prevalence

Sickle Cell Disease (SCD) and Thalassaemia are classified as the two main Haemoglobin Disorders, and in recent years have been acknowledged to have a global impact by the World Health Organisation (WHO). SCD comprises a group of inherited red blood cell conditions that result from the synthesis of variant or mutant haemoglobins. Over 300,000 babies are born worldwide with SCD mostly in low and middle income countries, with the majority of these births in Africa [1]. SCD originates in tropical regions as a result of its advantage against malaria. It is predominant among people from African, Asian, Arabian and Mediterranean countries; nonetheless it is a global health problem because of population migration. SCD results in early childhood death if left untreated, and its effect on the burden of health care is being recognised as a global issue in terms of chronic disease. Inheritance of a single sickle haemoglobin (HbS) gene results in a healthy sickle cell carrier state, while the inheritance of the HbS gene from both parents, or HbS with another variant haemoglobin gene (eg HbC, Hbβ-thalassaemia) results in symptomatic SCD.

Generally, the prevalence of healthy carriers (sickle cell trait) ranges between 10% and 40% across equatorial Africa and decreases to between 1% and 2% in Northern Africa and less than 1% in Southern Africa. In West African countries such as Ghana and Nigeria, the frequency of carriers is 15% to 30% while in East African countries such as Uganda and Tanzania it shows wide variations of up to 45% in some areas [2]. This distribution is thought to reflect current or historic exposure to plasmodium malaria infection as carriers appear to be protected from malaria associated deaths and thus have improved survival and therefore continued transmission of the HbS gene. The incidence of SCD at birth is determined by the prevalence of carriers in the population. SCD has remarkable public health implications for Africa. It contributes the equivalent of 5% to under-five deaths in Africa, with up to 16% in West Africa [1]. Thus in Nigeria, with an estimated carrier prevalence of 24%, 20 per 1000 births are estimated to be affected by SCD, resulting in 150,000 children with SCD born annually in Nigeria [2].

## Clinical Features and Medical Management

SCD is a chronic condition with recurrent episodes of pain and symptoms persistent throughout the lives of individuals. The clinical syndromes include anaemia, infection, and the consequences of blood vessel blockage (vaso-occlusion). The latter deprives tissues of oxygen and is suggested as the cause of the acute painful episodes, the hallmark of SCD, and other clinical syndromes such as stroke, chest complications, priapism, leg ulceration and chronic organ failure. The prominence of the pain is the basis upon which SCD has been named in certain cultures in West Africa [3]. These indigenous names are characterised by alliteration of letters that apparently signifies the persistence and recurrence of its painful symptoms. For example, the term 'Ahotutuo' is used by the 'Twi' people in Ghana to describe SCD; and the closest English translations would include 'body biting', 'body chewing', and 'beaten up' [3].

There is no universal cure for SCD and treatment options are rather limited, however improved knowledge has greatly advanced medical management over the past four decades. Antibiotic prophylaxis is used to prevent infections especially in children [4]. Other therapies aim to minimise the effects of symptoms of the disorder. Painful episodes (crises) are managed primarily with analgesia, and hydration [5]. Analgesic pain control is usually in progressive stages and requires a variety of medications ranging from paracetamol for mild pain to morphine for severe pain.

Blood transfusions may be required for stroke and other complications, and hydroxyurea has also been found to be very effective in reducing the 'sickling' process and consequently the frequency of pain and hospitalisations experienced by patients [5]. Bone marrow transplantation is a possible cure, however among other criteria, this requires a matched donor, and unfortunately is not feasible for all affected children [6].

## Psychosocial Impact

Psychosocial issues for people with SCD and their families mainly result from the impact of pain and symptoms on their daily lives, and society's attitudes to SCD and those affected. In Africa, cultural factors are particularly relevant to these problems because of beliefs and traditional practices. In Nigeria, beliefs are usually influenced by cultural and religious values, which influence health behaviour such as coping strategies. For example, among the Igbo communities, SCD is believed to be the result of malevolent 'Ogbanje' (reincarnation) that is repeated cycles of birth, death and reincarnation [7]. Other studies have shown that religious beliefs play a positive part in coping including prayer, faith in God and doctors, and a hopeful approach to health difficulties in Nigeria [8,9]. Previous research also revealed that compared with people with SCD living in the UK, those in Nigeria commonly used praying and hoping as an affective coping strategy, which seems to be influenced by external factors such as religion, faith in God, superstitions, and stigma [10].

The object of this study was to explore the psychosocial impact of SCD within a Nigerian population. It is acknowledged that psychosocial interventions for people with SCD are as important as medical treatment for comprehensive management.

## Methods

Pre-study focus groups were conducted earlier to explore and identify issues in general and specific concerns of individuals with SCD in order to develop or obtain appropriate measures to be used in the studies. These groups comprised of adolescents and adults aged thirteen years and over, were non-directive, but semi-structured with open-ended questions, and facilitation towards a goal at the end of each session. There were two group sessions with 6 to 8 participants, and each group talked about the same issues with the same set of questions. A number of open-ended questions were discussed in each group session, and answers were written down. The main areas explored included knowledge and understanding, beliefs, pain experience, coping, health service utilisation, social issues including education and employment, and quality of life. This led to the development of the questionnaire, which was subsequently used in the study. However, the questionnaire was not utilised in the focus group sessions.

### Measures

*Psychosocial Impact of Sickle Cell Disorder *- a self-complete questionnaire designed for the study (see Additional file 1). This is a 28-item questionnaire to examine: demographics, general perceptions, societal awareness, teachers and employers' attitudes, experiences with health services, emotional responses. The aim was to allow easy categorisation of responses, therefore 'Yes' and 'No' responses were used instead of 'Likert' scales for graded responses. This approach was adopted because of uncertainty about the literacy levels of some patients. Although there was no validation of the questionnaire, it was administered to a pilot sample of patients with SCD attending the National Sickle Cell Centre in Lagos, with no difficulties in completion expressed.

### Participants

Patients with SCD attending sickle cell outpatients' clinics in Lagos located at Lagos University Teaching Hospital, Gbagada General Hospital, Massey Street Children Hospital, and the National Sickle Cell Centre were recruited opportunistically to the study. They were all administered questionnaires during the clinic appointment by a team of two psychology interns and a research assistant over a two-month period. The average clinic attendance ranged from 30 to 45 at the three hospitals. None of the medical staff at the clinics were involved in the recruitment of patients to avoid coercion. The psychology interns and research assistant introduced the study in the waiting area with an opportunity for the participants to refuse consent, informing them that this would not affect their medical treatment.

## Results

Demographic characteristics of the participants are presented in Table 1. The total study sample was 408, which consisted of 194 males and 214 females. There were 155 (38%) adolescents aged 14 to18 years, and 253 (62%) adults aged 19 to 56 years.

**Table 1:** Demographic Characteristics of Patients Attending Sickle Cell Clinics in Lagos

Variable	Frequency
**Age Groups:**	
14-18	155 (38%)
19-23	108 (27%)
24-28	93 (23%)
29-33	28 (7%)
34-38	12 (3%)
39-43	6 (1%)
44-56	6 (1%)
	
**Gender:**	
Male	194 (48%)
Female	214 (52%)
	
**Ethnic Origin:**	
Hausa/Fulani	4 (1%)
Igbo	52 (13%)
Yoruba	305 (75%)
Others	47 (11%)
	
**Occupation:**	
Employed	60 (15%)
Student	311 (76%)
Housework	7 (2%)
Seeking Employment	25 (6%)
Retired	1 (0%)
Other	4 (1%)

All the adolescents were students who attended the Sickle Cell Clinic at Massey Street Children Hospital. The majority of all participants (75%) were from a Yoruba ethnic origin. The attitudes and perception about SCD were examined under three broad categories of general public, education and employment, and health services (Table 2). These were grouped into separate responses according to Adolescent and Adult ages.

**Table 2:** Responses to Attitudes and Perceptions about Sickle Cell Disorder

	Yes		No	
Category	Adolescents N = 155	Adults N = 253	Adolescents N = 155	Adults N = 253
**General Public:**				
Negative Societal Attitudes/Perception	96 (62%)	168 (66%)	59 (38%)	85 (34%)
Negative Approach by People	41 (27%)	93 (37%)	114 (73%)	160 (63%)
Need for More Awareness	132 (85%)	207 (82%)	23 (15%)	69 (18%)
Positive Effect of More Awareness	123 (79%)	198 (78%)	32 (21%)	55 (22%)
**Education and Employment:**				
Teasing/Bullying	29 (19%)	66 (26%)	126 (81%)	187 (74%)
Teacher/Staff Support	94 (61%)	128 (51%)	61 (39%)	125 (49%)
Employers Knowledge of SCD Status	0 (0%)	164 (40%)	0 (0%)	244 (60%)
Employers Discrimination	0 (0%)	77 (19%)	0 (0%)	331 (81%)
**Health Services:**				
Negative Attitudes	42 (27%)	79 (31%)	113 (73%)	174 (69%)
Good Understanding	144 (93%)	144 (57%)	11 (7%)	109 (43%)
Fair Treatment	103 (66%)	160 (63%)	52 (34%)	93 (37%)

The responses of the adolescents were mostly similar to the adults. The majority of participants thought that society in general had a negative image of SCD through its attitudes and perceptions, although they did not feel that people had a negative approach towards them because of their SCD. Nonetheless, a very large proportion of respondents thought that SCD as an illness is less known in society and needs more awareness, and if society was more aware of people with SCD it would be more positive about those with the condition. Participants had not experienced much teasing and bullying during their education in school, college or university. Discrimination from employers was also not widely reported. Experiences of health services were mostly positive especially among adolescents, as the vast majority of them felt that medical staff had a good understanding of SCD compared to adults. Despite their age group, most participants acknowledged that medical staff offered them fair treatment.

Emotional responses to SCD are presented in Table 3. Nearly half the number of participants indicated feelings of depression, however feelings of anxiety and self-hate were much less.

**Table 3:** Emotional Responses to Sickle Cell Disorder

Emotion	Frequency	
	Adolescents N = 155	Adults N = 253
Self-Hate	9 (6%)	21 (8%)
Depressive Feelings	84 (54%)	112 (44%)
Anxiety Feelings	11 (7%)	33 (13%)
All Emotions	7 (5%)	25 (10%)
None	44 (28%)	62 (25%)

## Discussion

The impact of chronic illness such as SCD on individuals may be grouped into a set of illness-related tasks: adjusting to the symptoms and incapacities; maintaining adequate relationships with health-care providers; and managing the emotional and social consequences of the illness [11]. The extent to which individuals are affected by a chronic illness may therefore be determined by their coping responses since dealing with its continuous demands requires the acquisition of new skills and modifications to daily life. Previous studies on psychosocial aspects of SCD generally examined the extent of its impact on children and adults, the ways in which affected families' function, and the resultant psychological adjustment. Some studies demonstrated that SCD is a risk factor for maladjustment (psychosocial functioning) in children and adolescents [12-15]. Earlier research also showed that rates of poor psychological adjustment of children with SCD remained relatively constant over time, however there was less stability in child psychological adjustment reported by children as opposed to reports by mothers [16].

This study sought to explore the psychosocial impact of SCD in a Nigerian population. The results support the notion that society's attitudes and perceptions had a psychosocial impact on people with SCD. Health beliefs could be influenced by external factors such as advice given by health workers, family support, and work responsibility. Beliefs can also be influenced by their culture. For example, it has been suggested that Nigerians have tried religious healing (prayer) as an alternative approach or in addition to routine medical treatment [17]. This may be due to causes of illness being attributed to "divine retribution" or the supernatural. There are cultural variations in concepts of health and illness relating to: views of illness, causes of ill health; acceptable treatment, types of treatment believed to be effective; reaction to symptoms, use of appropriate services. Within the Nigerian context, such beliefs lead to negative perceptions and attitudes about SCD.

About three-quarters of the study population were students, and over a third were adolescents aged 14 to 18 years. Anecdotal evidence suggests teasing and bullying are common complaints among school going children with SCD. This was reported in 23% of the study population. Other major psychosocial problems experienced by young people with SCD during their school going years have also been described in focus groups [18]. Important issues include fear of early death, fear of talking to friends and teachers about the condition, embarrassment about bedwetting and reluctance to take part in school trips because of this, teasing by colleagues due to jaundice and associated discolouration of their eyes, and anger should ill-informed staff consider the child as lazy and wanting to keep away from school activities. Anxieties that young people with SCD experience at school may result in the development of a negative image of themselves, teachers and school staff.

Mood is an important consequence of SCD. People with SCD commonly report low self-esteem and feelings of hopelessness as a result of frequent pain, hospitalisations, and loss of schooling (in children) and employment (in adults). These accounts could indicate depressive symptoms. Some studies have revealed anxiety and depression in children with SCD [19], and depression in adults [20]. Consistent with the literature, a considerable number the patients in this study had feelings of depression. Feelings of anxiety and self-hate were uncommon. Ohaeri and colleagues [8] in their work with 170 patients aged 11-20 years, in Nigeria, also noted that about 88% were worried and 55% has depressive thoughts about their condition.

There is considerable variability in the ability of people with SCD to cope with their condition. People with SCD experience different levels of health, and such variations can lead to differences in psychosocial functioning. Some people cope relatively well, attend school or work, and are active physically and socially. Others cope inadequately' leading more limited and secluded lives. Nonetheless, this may not necessarily be a consequence of severity of their condition, and the reasons for these should be sought and addressed. Quality of life in people with SCD may therefore be more impaired than that of the general population.

There are some limitations to the study. The use of dichotomous questions was mainly to address possible literacy problems in some patients who may have found it difficult to grade their responses on a Likert type scale, thus avoiding confusion. Dichotomous questions are useful in situations where the intention is to direct participants to express a clear opinion between opposing perspectives. However, a major drawback is that questions have only fixed alternatives, that is 'Yes' and 'No' in this case. Closed questions do not allow respondents to qualify or explain their answers, which may lead to bias in the interpretation due to the categories imposed. No standard questionnaires for the psychosocial impact of sickle cell disorder were identified from previous research, nor were there generic psychosocial impact measures in the chronic illness literature that could be employed for the purpose of this study.

Furthermore, information obtained was cross-sectional, longitudinal data would have enhanced the results. More information on socio-economic status would have been useful. Also, the opportunistic selection of patients in a clinic setting may have been biased; a community-based study may have yielded different results, given that the views of patients not receiving medical treatment could be different. Nonetheless, the study contributes to our understanding of the psychosocial impact of SCD within the African setting.

The findings of this exploratory study indicate that there is a need to develop appropriate psychosocial interventions within a global context. The aim should be more public education about SCD to change beliefs, attitudes, and stigma as a starting point. This should be followed by other interventions that are tailored depending on access to health care at different levels, and in different settings. In developing countries such as Nigeria, the focus would be initiating basic psychosocial interventions by non-specialised health workers in a primary care team at a Community Health Post (Level 1) or Primary Health Centre (Level 2), where psychologists or social workers are not be available. This would be in line with the WHO's current priority of primary care as a hub of coordination for person-centred care [21].

Within the global context, the aims of psychosocial interventions in chronic illnesses such as SCD are to help reduce negative thoughts and feelings about the condition, and encourage the acquisition or maintenance of coping strategies. In the case of SCD, psychological therapies should be offered as standard care in the management of SCD adjunctive to routine medical treatment, where studies of these therapies have shown encouraging results [22]. The overall goal is to help patients cope better, fulfil roles, and to achieve better quality of life. These interventions should be age-appropriate, and available in both hospital clinics and community-based settings depending on access.

## Competing interests

The authors declare that they have no competing interests.

## Authors' contributions

KA conceived, designed, and participated in the co-ordination of the study. FE participated in the co-ordination, data collection, and performed the statistical analyses. OO was involved in the design and supervision of the study. KA took the lead in the write up with support from FE, and review and editing by OO. All authors read and approved the final manuscript.

## Ethics approval

Ethics Approval was not sought for the study, however each individual had to give their consent for participation in the study.

## Supplementary Material

Additional file 1 **Psychosocial Impact of Sickle Cell Disorder**. A 29-item self-complete questionnaire designed for the study. Click here for file
