# Supplementary material for: Psychosocial impact of sickle cell disorder: perspectives from a Nigerian setting
Source: Global Health. 2010 Feb 20;6:2. doi: 10.1186/1744-8603-6-2 (PMC2836308; doi:10.1186/1744-8603-6-2)
Supplement: Additional file 1 — Psychosocial Impact of Sickle Cell Disorder. A 29-item self-complete questionnaire designed for the study. [file 1744-8603-6-2-S1.DOC]

# PSYCHOSOCIAL IMPACT OF SICKLE CELL DISEASE

**DEMOGRAPHICS:**

1. Gender

🞎 Male 🞎 Female

2. What is your age? ………………………

3. What is your ethnic origin?

🞎 Yoruba

🞎 Igbo

🞎 Hausa/Fulani

🞎 Other

4. What is your current living status?

🞎 Alone

🞎 With spouse/ partner

🞎 With parents

🞎 With other relatives

🞎 With unrelated persons

🞎 Other

5. What is your current marital status?

🞎 Never married

🞎 Separated

🞎 Widowed

🞎 Divorced

🞎 Married

6. What best describes your current main activity?

🞎 In employment, or self-employed

🞎 Retired

🞎 Housework

🞎 Student

🞎 Seeking work

🞎 Other, please specify………………….…………………………..

# PUBLIC ATTITUDES:

7. In general, do you feel that people see you in a negative way because of your sickle cell?

🞎 Yes

🞎 No

8. Have you ever felt that you have been treated unfairly or that your rights have been denied because of your sickle cell?

🞎 Yes

🞎 No

If ‘Yes’, how?

………………………………………………………………………………………………………………………………………………………………………………………………………………………………………………………………………

9. Have you ever experienced any negative attitudes due to any physical sickle cell complications you may have, for example, leg ulcers, or jaundice?

🞎 Yes

🞎 No

If ‘Yes’, please can you state what this physical complication was?

……………………………………………………………………………………………………………………………………………………………………………………………………………………………………………………………………....

10. Do you feel that there are more negative attitudes towards sickle cell because it largely affects black people?

🞎 Yes

🞎 No

11. Do you think that society has a negative image of sickle cell through its attitudes and perceptions?

🞎 Yes

🞎 No

12. Do you think that sickle cell disease as an illness is less known in society and needs more awareness?

🞎 Yes

🞎 No

13. If society was more aware of sickle cell disease, do you think that people would be more positive about sickle cell disease and those with sickle cell?

🞎 Yes

🞎 No

**EMPLOYMENT:**

14. Do you find it difficult to tell your employer(s) about your sickle cell?

🞎 Yes

🞎 No

If ‘Yes’, why is this?

🞎 I feel my capabilities will be underestimated

🞎 I fear that I will be viewed negatively

🞎Other, please specify……………………………………………………...

………………………………………………………………………………

15. Have you ever lost your job due to absenteeism as a result of sickle cell crises?

🞎 Yes

🞎 No

16. Have you ever been rejected from a job application because of your sickle cell?

🞎 Yes

🞎 No

17. Have you ever experienced discrimination in the work place as a result of your sickle cell?

🞎 Yes

🞎 No

If ‘Yes’ how?

………………………………………………………………………………………………………………………………………………………………………………………………………………………………………………………………………

**EDUCATION:**

18. Have you ever felt isolated from your peers at school/college/university because of your sickle cell?

🞎 Yes

🞎 No

19. Have you ever suffered from teasing or bullying at school/college/university because of your sickle cell?

🞎 Yes

🞎 No

20. Do you think that teachers and other staff are aware and supportive of your sickle cell?

🞎 Yes

🞎 No

**HEALTH SERVICES:**

21. Do you feel that the medical staff mistrust you or dismiss your sickle cell pain?

🞎 Yes, always

🞎 Yes, sometimes

🞎 No, never

22. Do you feel that you are labelled as a “drug seeker” or “drug addict” when seeking strong pain killers for your sickle pain?

🞎 Yes, always

🞎 Yes, sometimes

🞎 No, never

23. Do you feel that the hospital has a good understanding of SCD and provides you with good individual care?

🞎 Yes

🞎 No

24. When you are hospitalised, do you feel that you are treated fairly by medical staff compared to other patients?

🞎 Yes

🞎 No

**PSYCHOLOGICAL ISSUES:**

25. Do you ever avoid situations where you may be viewed unfavourably because of your sickle cell?

🞎 Yes

🞎 No

26. Do you avoid telling new friends about your sickle cell?

🞎 Yes

🞎 No

If ‘Yes’, why is this?

🞎 You’re worried about their reaction

🞎 You think they may judge you in a negative way

🞎 Other, please specify…………………………………………………..

……………………………………………………………………………

27. Do you find it difficult to keep relationships because of your sickle cell?

🞎 Yes

🞎 No

If ‘Yes’, why is this?

🞎 Other people do not seem to understand the illness

🞎 There are many complications of sickle cell that make it hard to keep friends

🞎 Other, please specify…………………………………………………

28. Have you ever experienced any of the following negative feelings as a result of your sickle cell? (Please tick all that apply)

🞎 Self-hate

🞎 Depression

🞎 Anxiety

THANK YOU FOR YOUR TIME
